# A comprehensive study on non-cancer-related mortality risk factors in elderly gastric cancer patients post-curative surgery

**DOI:** 10.1186/s12876-024-03170-6

**Published:** 2024-02-19

**Authors:** Yuki Ushimaru, Shinnosuke Nagano, Kazuhiro Nishikawa, Ryohei Kawabata, Tomohira Takeoka, Akihiro Kitagawa, Nobuyoshi Ohara, Hideo Tomihara, Sakae Maeda, Mitsunobu Imazato, Shingo Noura, Atsushi Miyamoto

**Affiliations:** Department of Gastroenterological Surgery, Sakai City Medical Center, 1-1-1 Ebaraji-Cho, Nishi-Ku, Sakai City, Osaka, 593-8304 Japan

**Keywords:** Gastric cancer, Non-cancer related death, Elderly patients

## Abstract

**Background:**

The increasing incidence of gastric cancer in the elderly underscores the need for an in-depth understanding of the challenges and risks associated with surgical interventions in this demographic. This study aims to investigate the risk factors and prognostic indicators for non-cancer-related mortality following curative surgery in elderly gastric cancer patients.

**Methods:**

This retrospective analysis examined 684 patients with pathological Stage I—III gastric cancer who underwent curative resection between January 2012 and December 2021. The study focused on patients aged 70 years and above, evaluating various clinical and pathological variables. Univariate analysis was utilized to identify potential risk factors with to non-cancer-related mortality and to access prognostic outcomes.

**Results:**

Out of the initial 684 patients, 244 elderly patients were included in the analysis, with 33 succumbing to non-cancer-related causes. Univariate analysis identified advanced age (≥ 80 years), low body mass index (BMI) (< 18.5), high Charlson Comorbidity Index (CCI), and the presence of overall surgical complications as significant potential risk factors for non-cancer related mortality. These factors also correlated with poorer overall survival and prognosis. The most common cause of non-cancer-related deaths were respiratory issues and heart failure.

**Conclusion:**

In elderly gastric cancer patients, managing advanced age, low BMI, high CCI, and minimizing postoperative complications are essential for reducing non-cancer-related mortality following curative surgery.

## Introduction

Recent years have seen a notable increase in gastric cancer among the elderly [1–3]. With the global population aging, the incidence of this disease in the elderly is expected to rise further. This situation calls for a detailed examination of surgical interventions appropriate for this demographic. Managing gastric cancer in the elderly is complex, requiring a balance between effective treatment and accommodation the unique physiological and comorbid conditions of the elderly. Additionally, the typical male predominance and less aggressive nature of gastric cancer in this age group add to the complexity of management [4].

Gastric cancer surgery, critical curative intent, presents significant challenges, particularly for the elderly. Surgical procedures can compromise gastric and intestinal functions, leading to postoperative complications that are especially harmful in the elderly [3, 5, 6]. The capacity of older patients to withstand the stress of surgery is crucial; even a successful curative resection can lead to deteriorations in health and quality of life.

A major concern in the post-surgical management of elderly gastric cancer patients is the incidence of non-cancer-related fatalities. Previous study have identified key preoperative factors like sex, neutrophil-to-lymphocyte ratio, and skeletal muscle mass index as significant risk factors for non-cancer-related death in this patient group [7]. Despite these insights, a considerable number of elderly patients undergoing curative resection still succumb to non-cancer-related fatalities [8–10]. This highlights the importance of a comprehensive understanding of the multifaceted challenges these patients face, emphasize the need to further investigate and address these challenges.

Given this context, our study aims to deepen the understanding of non-cancer-related mortality in postoperative elderly gastric cancer patients. We seek to expand upon existing research by exploring additional or interrelated factors, with the goal of uncovering nuances that have been previously overlooked. Our findings are intended to guide clinical decisions and shed light on potential interventions that could mitigate these risks, thereby enhancing postoperative care and outcomes for elderly gastric cancer patients.

## Patients and methods

### Patients

This study, anchored at the Sakai City Medical Center, drew from a prospectively assembled database. From January 2012 to December 2021, 684 patients diagnosed with pathological Stage I—III gastric cancer underwent curative resection. The surgical procedures encompassed Distal Gastrectomy (DG), Proximal Gastrectomy (PG), and Total Gastrectomy (TG). Surgical techniques included both open surgery (OS) and Minimally Invasive Surgery (MIS)—comprising laparoscopic and robotic surgery methods. We zeroed in on elderly patients aged 70 years and above for this study. Exclusions were made for patients who experienced recurrence, those who did not achieve curative resection, those who succumbed to other cancers, and those with atypical histological types. All cases included were histologically verified as gastric cancer. The TNM classification adhered to the guidelines set by the Japanese Gastric Cancer Association [11, 12]. All treatments were dispensed in line with the Japanese Gastric Cancer Treatment Guidelines [10, 13].

Data for the chosen cases was collated from the aforementioned prospective database for analysis. The primary variables under scrutiny included age, gender, body mass index (BMI), Charlson Comorbidity Index (CCI), American Society of Anesthesiologists physical status (ASA-PS), modified Glasgow Prognostic Score (mGPS), Prognostic Nutritional Index (PNI), Neutrophil–lymphocyte ratio (NLR), and other patient backgrounds. Surgical approach (OS / MIS approach), surgical procedure (TG / non-TG (DG, PG)), overall surgical complications (Present / Absent), histological classification (undifferentiated / differentiated), pathological T status (T2-4 / T1), pathological N status (N1-3 / N0), and pStage (I / II / III) were also taken into account. For patients who passed away due to other diseases, the cause of death was documented. The non-gastric cancer-related death group was juxtaposed with a counterpart group to discern significant disparities in the aforementioned variables. Both univariate and multivariate analyses were undertaken to identify potential risk factors tied to non-cancer-related mortality.

In this study, we carefully defined the follow-up period for patients who survived without recurrence, typically observing them for 5 years post-surgery as part of our surveillance protocol. Additionally, there were cases where follow-up extended beyond 5 years as an option. This timeframe was chosen to more accurately assess non-cancer-related mortality while minimizing the inclusion of patients who might develop recurrence. Patients lost to follow-up within the first year post-surgery or who experienced recurrence within the 5-year period were excluded.

### Statistical analysis

Non-gastric-cancer specific survival was delineated as the duration from the surgery date to the date of death attributed to non-gastric-cancer. Survival rates were computed using the Kaplan–Meier method and contrasted using the log-rank test. Clinicopathological characteristics of the two groups were compared using the chi-squared test for categorical variables and the Mann–Whitney U test for continuous variables. The Cox proportional hazards model ascertained the hazard ratio (HR) of variables on non-gastric-cancer specific survival in univariate analyses with forest plot, adjusting for factors like age, gender, BMI, CCI, surgical approach, surgical procedure, pathological TNM status, histological classification, mGPS, and NLR. A *p*-value below 0.05 was deemed statistically significant. All statistical evaluations were conducted using JMP® PRO software (JMP version 16.1.0, SAS Institute, Cary, NC).

## Results

### Patient characteristics and details of non-cancer related deaths

**Fig. 1:**
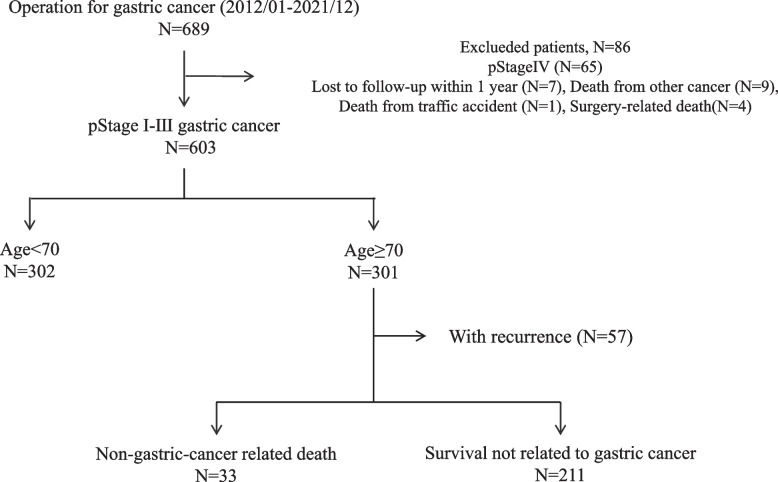
Study flow chart. Illustrates the patient selection from initial diagnosis to final analysis, detailing exclusions and the final cohort analyzed

A total of 689 patients were initially considered for this study. Of these, 85 patients were excluded for various reasons: 7 were lost to follow-up within a year, 9 died of other cancers, 1 died in a traffic accident, 4 died due to surgery-related causes, and 65 were diagnosed with Stage IV gastric cancer (including one overlapping case). This resulted in 603 patients with diagnosed pathological Stage I-III underwent curative resection. The focus group for further detailed analysis comprised 301 patients aged 70 or above. Post the exclusion of 61 patients due to recurrence, 244 patients were analyzed, with 33 succumbing to non-cancer-related causes (Fig. 1). Notably, respiratory-related issues (36.4%), and heart failure (24.2%) were leading non-cancer mortality factors (Table 1). The median age at death was 82 years, spanning from 73 to 93 years. The median interval from surgery to death stood at 705 days, ranging between 42 to 1740 days.

**Table 1:** Breakdown of other causes of death and the details

Cause of Death	*N* = 33
Respiratory associated causes	20 (60.6%)
Heart Failure	8 (24.2%)
Senility (or Natural causes)	4 (12.1%)
Kidney Failure	1 (3.0%)
Age at the time of death	82 years old (73 – 93 years old)
Time period from surgery to death	705 days (42 – 1740 days)

A comparative analysis revealed significant differences in characteristics between patients who survived without recurrence and those who died from non-cancer related causes (Table 2). The non-cancer-related death group had a higher median age and a lower median BMI than the survivors. Additionally, the deceased had more elevated CCI scores and a higher incidence of surgical complications.

**Table 2:** Patients’ characteristics

	Alive without recurrence *N* = 211	Death from non-cancer related cause *N* = 33	*P* value
Age	77 (70–95)	80 (71–89)	0.015
Gender			0.41
Male	145 (68.7%)	25 (75.8%)	
Female	66 (31.3%)	8 (24.2%)	
BMI	22.8 (15.3 – 33.3)	20.9 (14.7—34.2)	< 0.001
Charlson Comorbidity Index			0.003
Low	96 (45.5%)	6 (18.2%)	
Medium	90 (42.7%)	17 (51.5%)	
High	24 (11.4%)	8 (24.2%)	
Very high	1 (0%)	2 (6.1%)	
ASA-PS			0.29
1	10 (5.0%)	0 (0%)	
2	150 (74.6%)	25 (75.8%)	
3	39 (19.4%)	8 (24.2%)	
4	2 (1.0%	0 (0%)	
Modified GPS			0.37
0	158 (74.9%)	21 (63.6%)	
1	36 (17.1%)	9 (27.3%)	
2	17 (8.1%)	3 (9.1%)	
PNI	48.1 (24.5 – 64.2)	45.6 (29.7 – 56.6)	0.051
NLR	2.37 (0.76 – 16.14)	2.55 (1.11 – 16.0)	0.08
Surgical approach			0.23
Open surgery	85 (40.3%)	17 (51.5%)	
Minimal invasive surgery	126 (59.7%)	16 (48.5%)	
Surgical procedure			0.84
TG	48 (22.8%)	7 (21.2%)	
Non-TG (DG / PG)	163 (77.3%)	26 (78.8%)	
Overall surgical complications			0.021
Presence	59 (27.8%)	16 (48.5%)	
Absence	152 (72.0%)	17 (51.5%)	
Histological classification			0.27
Undifferentiated	71 (33.7%)	8 (24.2%)	
Differentiated	140 (66.4%)	25 (75.8%)	
Pathological T status			0.75
T2-4	96 (45.5%)	16 (48.5%)	
T1	115 (54.5%)	17 (51.5%)	
Pathological N status			0.12
N1-3	58 (28.6%)	14 (42.4%)	
N0	145 (71.4%)	19 (57.6%)	
Pathological Stage			0.54
I	128 (61.1%)	16 (48.5%)	
II	56 (26.5%)	11 (33.3%)	
III	26 (12.3%)	6 (18.2%)	
Adjuvant chemotherapy			0.42
Presence	37 (17.5%)	4 (12.1%)	
Absence	174 (82.5%)	29 (87.9%)	

Values are presented as median (range) (*) or number (%). *P* = 0.05 was considered statistically significant

*BMI* Body mass index, *CCI* Charlson Comorbidity Index, *ASA-PS* American Society of Anesthesiologists physical status, *mGPS* Modified Glasgow Prognostic Score, *PNI* Prognostic Nutritional Index, *NLR* Neutrophil–lymphocyte ratio, *DG* Distal Gastrectomy, *PG* Proximal Gastrectomy, *TG* Total Gastrectomy

### Evaluation of risk factors for non-cancer related deaths after gastric cancer surgery

**Fig. 2:**
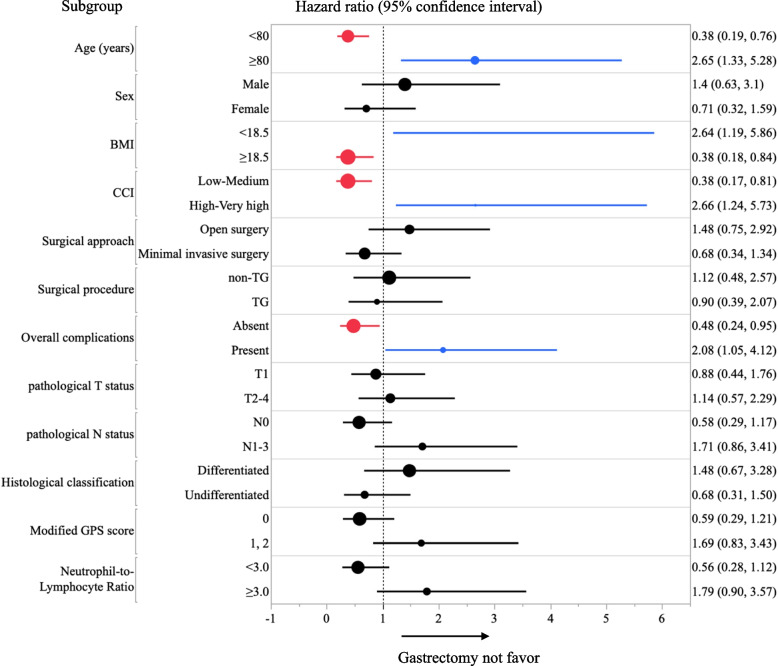
Forest plot—Other-cause mortality hazard risk

Univariate analysis identified several risk factors for non-cancer-related nirtality (Fig. 2). Advanced age (≥ 80 years), high CCI score (high / very-high) and lower BMI (< 18.5) significantly increased risk. Upon further analysis, it was observed that the association between the severity of postoperative complications, as classified by Clavien-Dindo (C-D) grading system, and non-cancer-related deaths varied. Specifically, for complications graded as C-D ≥ I, there was a significant correlation with non-cancer-related mortality (hazard ratio 2.08, 95% CI: 1.05 – 4.12). However, when considering higher-grade complications, this correlation became less apparent. For C-D ≥ II complications, the hazard ratio was 1.94 (95% CI: 0.98 – 3.84), and for C-D ≥ III, it was 0.91 (95% CI: 0.32 – 2.60). It is important to note that the limited number of cases, particularly for higher-grade complications, may have impacted the statistical power of our analysis.

### Survival curves for evaluated risk factors

**Fig. 3:**
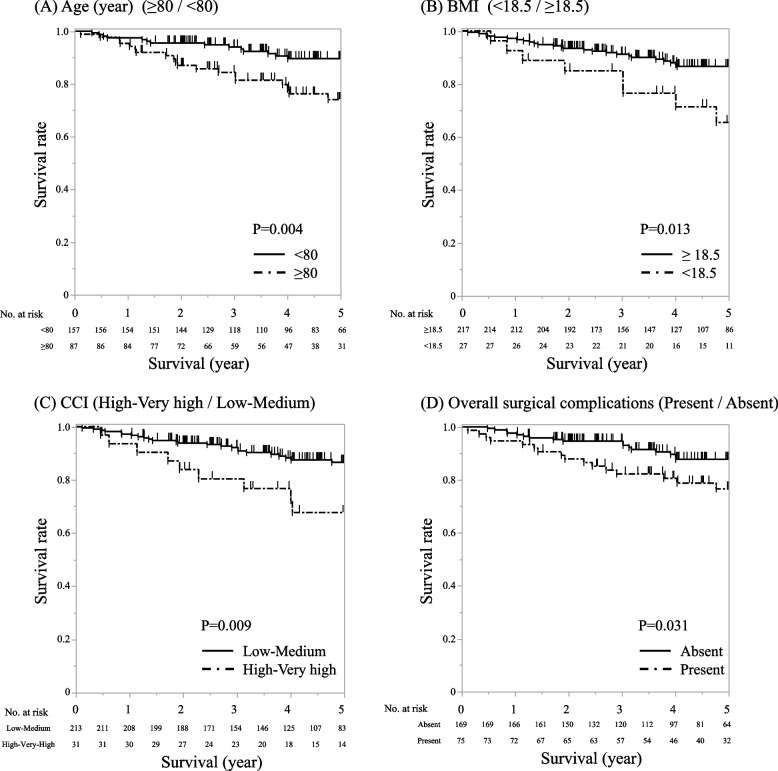
Survival Curves for Identified Risk Factors in Elderly Gastric Cancer Patients Post-Curative Surgery. **A** Survival rates of patients aged ≥ 80 years vs. those aged < 80 years. **B** Survival rates based on Body Mass Index (BMI) categories. **C** Survival rates based on Charlson Comorbidity Index (CCI). **D** Impact of postoperative complications on survival rates

Kaplan–Meier survival curves highlighted significant survival disparities based on age, BMI, CCI, and postoperative complications (Fig. 3). Patients over 80 years (*p* = 0.004), those with a BMI under 18.5 (*p* = 0.013), and those with higher CCI scores (*p *= 0.009) exhibited notably poorer survival rates. Similarly, the occurrence of postoperative complications was associated with reduced survival (*p* = 0.031).

## Discussion

The escalating incidence of gastric cancer among the elderly, reflective of broader demographic shifts towards an aging population, presents unique clinical challenges [1–3]. This demographic shift not only intensifies the complexities inherent in managing elderly patients but also necessities a nuanced understanding of the multifactorial nature of their health outcomes, particularly the elevated incidence of non-cancer-related mortality post-surgery [2, 7–9]. Previous research has identified individual risk factors such as sex, neutrophil-to-lymphocyte ratio, and skeletal muscle mass index [6], but did not fully address their collective impact on mortality. Our study extends this knowledge by evaluating a broader range of factors, including age, BMI, CCI, and postoperative complications. We found that advanced age (≥ 80 years), high CCI score, and lower BMI (< 18.5) significantly elevate the risk of non-cancer-related mortality. This highlights the importance of a comprehensive risk assessment, considering not only cancer-related factors but also a wider spectrum of health issues [14]. Our findings challenge existing paradigms and emphasize the need for tailored postoperative management strategies in elderly gastric cancer patients, aiming to improve overall outcomes by addressing both cancer and non-cancer-related health risks.

The adoption of MIS in gastric cancer treatment, particularly for elderly patients, represents a paradigm shift in surgical practice [15, 16]. While its less invasive nature is associated with reduced postoperative complications and expedited recovery, the implications of MIS on long-term outcomes, such as non-cancer-related mortality, are less clear. Furthermore, research indicates that the outcomes of MIS in elderly patients are comparable to those in middle-aged patients, emphasizing its efficacy regardless of age [17]. The benefits of MIS, such as diminished postoperative pain and shorter hospital stays, make it a promising approach for treating advanced gastric cancer in the elderly population [18]. Our findings suggest that, despite the recognized perioperative benefits of MIS, including enhanced safety compared to traditional open surgery (OS), its influence on non-cancer-related mortality in the elderly is not as pronounced as previously thought [19]. This observation necessitates a reevaluation of the long-term outcomes associated with MIS, especially in the context of an aging patient population. While the reasons behind this observation remain unclear in our study, it suggests a need for further investigation using national-scale databases to understand the full implications of MIS in this context.

The Comprehensive Geriatric Assessment (CGA) has been recognized as an invaluable tool in the evaluation and management of elderly cancer patients, providing a multidimensional diagnostic process to assess medical, psychosocial, and functional capabilities [20–22]. This assessment is crucial for formulating coordinated treatment and follow-up plans. Recent evidence suggests that CGA can predict postoperative complications and survival in elderly cancer patients [23, 24], and interventions based on CGA findings have been shown to enhance outcomes in this demographic [25]. In the context of our study, while we do not directly assert the application of CGA, we propose that it could be an essential tool in the preoperative evaluation, particularly for identifying risk factors such as low BMI and high CCI scores. By integrating CGA into the preoperative assessment, there is a potential to improve surgical preparation and optimize care, especially for those patients with higher risks, potentially leading to better postoperative outcomes.

Intriguingly, our data indicated that the TNM classification, traditionally a robust prognostic factor, did not emerge as an independent determinant in our cohort. This observation might be ascribed to the selection bias of excluding initial cases, thereby sidelining tumor factors [26]. This revelation implies that in elderly patients who have undergone curative resection, other determinants, such as comorbidities and overall health status, might play a more pivotal role in shaping outcomes than the tumor's attributes.

Given the rising prevalence of gastric cancer in the elderly and the unique challenges they pose, a paradigm shift in management is called for. Tailored treatment plans that take into account not just the tumor attributes but also the patient's overall health status, functional reserves, and preferences are indispensable [27]. Multidisciplinary teams, comprising geriatricians, nutritionists, and rehabilitation specialists, should be integral to the care of these patients. Moreover, postoperative monitoring and interventions should zero in on preventing and managing non-cancer-related complications.

This study, while offering crucial insights into the non-cancer-related mortality in elderly gastric cancer patients, has several limitations warranting consideration. Foremost, being derived from a single-institution database, it may not fully represent the broader elderly patient population, limiting the generalizability of our findings. The sample size, although substantial for this specific setting, remains modest, particularly in terms of the number of non-cancer-related death events, which could affect the robustness of our conclusions. The study's retrospective design inherently carries the risk of biases in data collection and interpretation. Socioeconomic factors, which can significantly impact patient outcomes, were not included in our analysis. Furthermore, while patients with concurrent cancers were excluded, the potential influence of their prior treatments or interventions was not assessed. It is also noteworthy that our focus on elderly patients was intentional to explore specific challenges in this group, and thus, a younger patient control group was not deemed necessary for our study objectives. To address these limitations and validate our findings, future research should employ larger, multi-institutional datasets, possibly integrating a wider array of demographic and socioeconomic variables.

In conclusion, this study provides critical insights into the factors contributing to non-cancer-related mortality in elderly gastric cancer patients. Our findings underscore that advanced age, low BMI, high CCI, and overall postoperative complications significantly influence patient outcomes. This revelation highlights the need for a holistic approach in the management of these patients, wherein a comprehensive risk assessment should be considered, encompassing both cancer-related and broader health-related factors. Our research suggests that tailored postoperative management, which includes vigilant monitoring and management of complications, is imperative in improving survival outcomes. The development of such strategies should be underpinned by a multidisciplinary approach, taking into account the overall health status and individual preferences of the elderly patient population.

## Data Availability

No datasets were generated or analysed during the current study.
